# Non-*Candida* mycosis in Gulf Cooperation Council (GCC) countries: perspective of a low-incidence region

**DOI:** 10.1186/s12879-025-10680-5

**Published:** 2025-02-23

**Authors:** Abdullah AlSaleh, Mohammed Shahid

**Affiliations:** 1 Microbiology, Immunology and Infectious Diseases Dept., College of Medicine and Health Sciences, Arabian Gulf University, Manama, Kingdom of Bahrain; 2 Occupational Health Directorate, Ministry of Health, Kuwait City, Kuwait

**Keywords:** Mycosis, Invasive, Molds, Fungal infections, Gulf Cooperation Council

## Abstract

**Background:**

Fungal pathogens are ubiquitous microorganisms that are implicated in a wide range of infections, affecting individuals with underlying health conditions and immune suppression therapy; however, immunocompetent individuals may also be at risk. Among these infections, many are caused by molds and yeasts other than *Candida* and are recognized in clinical practice, such as aspergillosis, mucormycosis, fusariosis, phaeohyphomycosis, and basidiobolomycosis, among others, each presents different clinical manifestations and requires clinical management specific to the site of involvement. Although pathogenic fungal contaminants and potential sources of mycosis in humans are plentiful in Gulf Cooperation Council (GCC) countries, epidemiological reports regarding mycosis in the region are scarce.

**Aim:**

The aim of this review is to shed some light on the epidemiology of clinically associated molds and yeasts other than *Candida* and to survey all related case reports and epidemiological studies conducted in the GCC over the past 10 years.

**Methods:**

A comprehensive search of the Medline (PubMed) and Scopus databases was conducted using the following keywords: Aspergillosis, Mycosis, Mucormycosis, Fusarium, Kuwait, Bahrain, Saudi Arabia, Qatar, Oman and the United Arab Emirates. A timeframe was set to include only articles that were published from 2014 to 2024.

**Results:**

One hundred thirty-five of the 1563 articles examined fulfilled the purpose of this review. Most studies were in Saudi Arabia (45%), Qatar (18%) and Kuwait (16%). Mucormycosis, aspergillosis, phaeohyphomycosis and basidiobolomycosis were among the most commonly reported fungal infections in the GCC, with corresponding mortality rates of 53%, 37%, 69% and 24%, respectively. The average estimations of non-*Candida* fungal infections indicate a low regional incidence in comparison with global estimations.

**Conclusion:**

Awareness and a high index of suspicion are warranted in successfully managing non-*Candida* mycosis. More specific immunological and molecular markers are needed for differential diagnosis to rule out fungal infections. Additionally, incorporating non-*Candida* mycosis-related antifungal resistance surveys in GCC national surveillance efforts should be enforced, especially when considering the increase in global mycosis rates.

## Introduction

Fungal pathogens are ubiquitous unicellular or multicellular microorganisms that are implicated in a wide range of indolent, recalcitrant and acute infections. They are associated with localized, disseminated, and systemic mycosis that affects mainly individuals with underlying health conditions such as diabetes mellitus, cancer, chronic pulmonary disorders, and immune suppression therapy; however, immunocompetent individuals may also be at risk. The global burden of serious fungal infections is estimated to surpass 150 million established cases, with an estimated incidence of 6.55 million cases annually [1, 2]. Among these infections, many are caused by molds and yeasts other than *Candida*; for example, the global annual incidence of invasive aspergillosis and mucormycosis has reached more than 2 million and 200,000, respectively [2]. In fact, the crude mortality of non-*Candida* mycosis is estimated to reach more than 2 million deaths annually [2]. In line with the unprecedented emergence of mycosis worldwide, the World Health Organization (WHO) has developed a Fungal Priority Pathogen List (FPPL) that categorizes 19 fungal pathogens into three priority groups, namely, critical, high, and medium, on the basis of the severity of infection and their significant role in public health [3]. The list focuses on systemic invasive infections only, yet superficial and mucosal infections could be added in the future [3].

Fungi, the causative agents of mycosis, are capable of growing on most organic and inorganic materials and surviving in a plethora of biotic and abiotic environments; in fact, they are widely considered to constitute the largest spectrum of host ranges of any pathogen [4, 5]. Many fungal pathogens also have the potential to undergo horizontal gene transfer, genetic recombination and hybridization, moving virulence genes among clonal lineages and consequently enabling the formation of novel pathogenic clones [6]. This remarkable multifariousness is largely attributed to resilient expansive dispersibility accompanied by the ability to survive outside a host, existing as living saprophytes or durable spores [5]. Indeed, fungi comprise most of the viable bioaerosols in the air, with human breath containing an average range of 1–10 fungal spores [7]. Notably, exposure to components of these organisms or their secondary metabolites, such as mycotoxins, is associated with skin irritation, allergic reactions, pneumonitis and the onset of infections [4].

Furthermore, fungal existence in relation to humans may be presented as asymptomatic uncommon colonizers in human infections, opportunistic agents with advantageous predispositions in human infections, or highly virulent systemic pathogens [8]. The variety in fungal pathology is attributed to differences in pathogenicity and virulence factors, even between closely related fungal genera [8]. For instance, the production of cell wall-bound melanin pigments in dematiaceous molds and black yeasts facilitates immune cell evasion and the neutralization of oxidative stress [8, 9]. Similarly, the production of chitin, mannan polysaccharides, the carotenoids torulene and torularhodin play important roles in cellular development, inhibiting phagocytosis and maintaining the composition of the cell wall [10–12]. Moreover, biofilm formation is another virulence factor that facilitates the withstand of antifungal agents and severe environmental conditions as well as the induction of fungal biofilm-mediated infections such as osteoarticular mycosis [13, 14]. The expression of antifungal resistance phenotypes is another major determinant of fungal pathogenicity [15]. Antifungal resistance may be established through morphism at the target drug site, overexpression of efflux pumps preventing the accumulation of antifungals, or intrinsic resistance to specific agents, such as azole resistance, in *Fusarium* sp [15, 16].

Despite the relative infrequency of some non-*Candida* fungal infections, managing severe and invasive mycosis is a challenging venture. The inaccessibility of appropriate therapeutic agents, discrepancy in antifungal tolerance, limited differential diagnostic tests and abundance of associated risk factors are some of the challenges hindering the quality management of non-*Candida* mycosis [3]. Additionally, the concomitant occurrence of mycosis with bacterial and viral infections manifests potential complications in treatment in relation to drug‒drug interactions, toxicity and the immunosuppressive effects of some agents, resulting in antifungal therapy failure [17, 18].

One major challenge in managing non-*Candida* mycosis is the change in the nomenclature of fungal pathogens, which complicates proper etiologic agent identification and, in turn, compromises quality treatment [19]. Recently, the classification criteria have shifted from phenotypic to genotypic considerations, affecting many previously established phylogenetic relationships and morphological categories [20]. Consequently, limiting the use of many reference mycological textbooks, encumbering interpreting and equating past data for future reports, not to mention, the toll on laboratories that are unable to adapt molecular identification methods [20, 21]. Considering the aforementioned shortcomings, it is widely believed that relying on molecular classification could potentially provide a more robust method for the identification of fungal pathogens [22].

Despite the grave health burden associated with fungal infections, there is a knowledge gap in the status of non-*Candida* mycosis in Gulf Cooperation Council (GCC) countries. Hence, the aim of this review is to shed some light on the epidemiology of clinically associated molds and yeasts other than *Candida* in the GCC.

## Methods

**Fig. 1:**
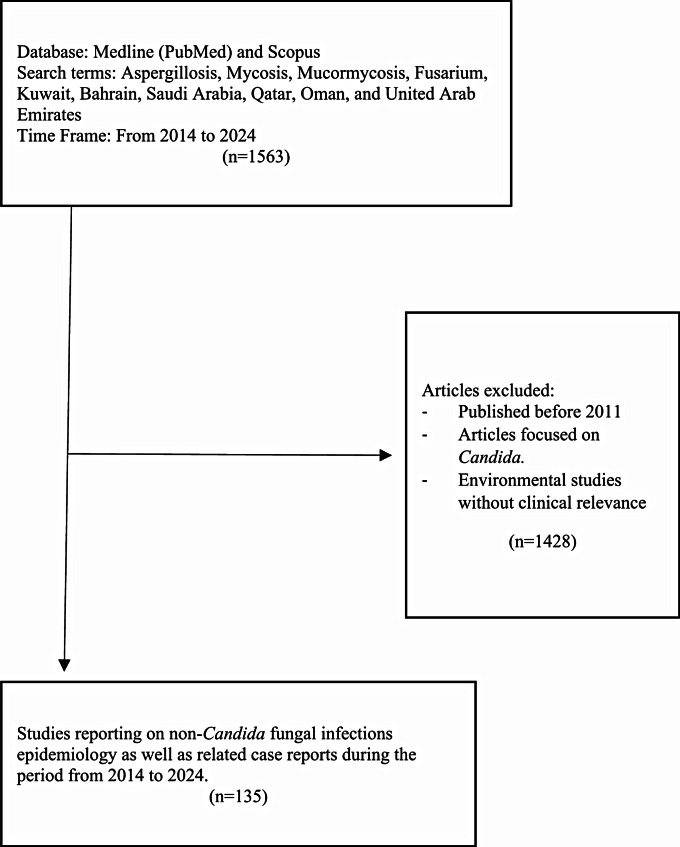
Article inclusion and exclusion process for this review

In this narrative review, a comprehensive search of the Medline (PubMed) and Scopus databases was conducted using the following key words: aspergillosis, mycosis, mucormycosis, fusarium, Kuwait, Bahrain, Saudi Arabia, Qatar, Oman, and the United Arab Emirates. A timeframe was set to include only articles that were published from 2014 to 2024. Case reports in addition to retrospective and prospective epidemiology studies were included in this study. Articles focused mainly on candidal infections, or the environmental impact of fungi were excluded. One hundred thirty-five out of 1563 articles fulfilled the purpose of this review (Fig. 1). Most of the articles included in this work were from Saudi Arabia (45%), Qatar (18%), Kuwait (16%) and Oman (14%), whereas studies from Bahrain and the UAE were rare.

## Results and discussion

### Regional perspective

Mycosis is a term that encompasses various phenotypes of infections caused by molds and yeasts, ranging from superficial dermatophytosis to fatal visceral invasive infections. The spectrum of conditions often depends on underlying comorbidities that may include cancer, solid organ transplantation, immunosuppressive therapy, mechanical ventilation, uncontrolled diabetes mellitus, asthma and chronic obstructive pulmonary disorder (COPD), among others [3, 23]. Many forms of non-*Candida* mycosis are recognized in clinical practice, such as aspergillosis, mucormycosis, fusariosis, phaeohyphomycosis and basidiobolomycosis, among others, each presents different clinical manifestations and requires clinical management specific to the site of involvement [19, 24–26]. Although pathogenic fungal contaminants and potential sources of mycosis in humans are often dispersed in indoor and outdoor environments in GCC countries, epidemiological reports concerning mycosis in the region are scarce, often only concentrating on *Candida sp*. pathophysiology, which is a sentiment shared with a previous regional review on the burden of invasive fungal infections in the Arab world, the Middle East and North Africa (MENA) region [27].

GCC countries share a relatively similar climate, characterized by arid hot summers and moderate winters; however, some variations may occur among these countries, especially in terms of the rates of humidity and dust precipitation. For example, dust storms occur more frequently in countries such as Kuwait and Saudi Arabia, with the annual average dust precipitation rate reaching 255 days per year in Kuwait [28]. Whereas countries such as Qatar and the Kingdom of Bahrain sustain an average high relative humidity of 70% and 83%, respectively [29, 30]. Indeed, desert soil, sandstorms and humidity are recognized as hospitable conditions for fungal growth and dissemination, serving as potential sources of mycosis in humans [5, 31]. In an effort to survey fungal communities in desert soil throughout 12 locations in the Arabian Peninsula, the most common genera isolated were found to include clinically relevant fungi such as *Aspergillus*, *Alternaria*, *Fusarium* and *Penicillium* [32]. Even when sampling desert soils with high salinity, mycosis-associated genera were predominant [33]. Owing to the high rate of dust precipitation in the region, pathogenic fungal genera have been consistently isolated in both indoor and outdoor environments. In a taxonomic characterization study on outdoor air in Kuwait, remote and urban areas were sampled over three seasons, and fungal genera associated with mycosis and allergies, such as *Cryptococcus*, *Alternaria*, *Aspergillus*, and *Candida*, among others, were regularly isolated throughout the study period, with insignificant variations in terms of location and season [34]. Interestingly, indoor air sampling studies in Kuwait also, reported a substantial prevalence of mycosis-associated genera; in fact, several *Aspergillus* species with triazole resistance or reduced susceptibility have been isolated across many tertiary hospitals [35–37]. Similar findings were reported in another effort to investigate outdoor airborne fungi in Qatar, with slight seasonal variations across the year; additionally, the report revealed no significant difference in fungal population composition between industrial and urban areas [29]. Furthermore, high humidity is also associated with fungal growth, especially skin infections [11]. In a 4-year retrospective study regarding skin conditions in Oman, it was reported that 41% of all skin conditions were due to fungal infections, many of which were caused by dermatophytes, and these infections were attributed to hot and humid weather [38].

The scarcity of epidemiological reports and the perceived regional low incidence of non-*Candida* mycosis may lead to it being overlooked in clinical care [1]. These infections often cause nonspecific symptoms in patients and may be mistakenly diagnosed as something else, despite the attainable quality health care and the availability of therapeutic options in GCC countries [1, 27, 39]. In fact, early diagnosis is paramount in obtaining a favorable prognosis for non-*Candida* mycosis patients, so appropriate management can be initiated; thus, a higher index of suspicion is needed in clinical care [25].

In an effort to gauge the epidemiology of non-*Candida* mycosis, we surveyed all related case reports and epidemiological studies conducted in the GCC in the past 10 years (2014–2024). Mucormycosis, aspergillosis and phaeohyphomycosis were among the most common reported infections, as shown in Table 1. Further elaboration on the status of non-*Candida* mycosis in the GCC follows below.

## Common mycosis forms

### Aspergillosis

Aspergillosis is a heterogeneous infection caused by the fungus *Aspergillus* sp.; it can affect immunocompromised as well as immunocompetent individuals with a variety of phenotypes [24]. Many forms of aspergillosis have been reported in clinical practice; for example, it can have an invasive nature like Invasive Aspergillosis (IP) pulmonary, rhino-orbital or disseminated phenotypes, which often result in high morbidity with *Aspergillus* osteomyelitis complications [14, 40]. Other invasive forms include Chronic Necrotizing Pulmonary Aspergillosis (CNPA), Chronic Cavitary Pulmonary Aspergillosis (CCPA) and Chronic Fibrosing Pulmonary Aspergillosis (CFPA) [40]. Interestingly, these many different forms of IA are reported to be involved in approximately 75% of invasive mold infections (IMIs) in children globally [18]. Noninvasive presentations are also reported, such as pulmonary, renal, or cardiac aspergilloma, as well as some allergic forms, such as Allergic Bronchopulmonary Aspergillosis (ABPA), Allergic Fungal Sinusitis (AFS) and Severe Asthma with Fungal Sensitization (SAFS) syndrome, but these disease presentations are often indolent and difficult to diagnose, mainly because of the convoluted diagnostic criteria for each phenotype [24, 40].

The most commonly reported etiologic agent worldwide is *Aspergillus fumigatus*, but other species, such as *A. flavus*, *A. terreus*, *A. niger*, and *A. versicolor*, have also been reported [41]. In GCC countries, a similar distribution has been reported, as 53% of IA cases in Bahrain were caused by *A. fumigatus*, followed by *A. niger* and *A. flavus* at 28% and 12%, respectively. Additionally, 60% of invasive *Aspergillus* infections were caused by *A. fumigatus*, as reported in a collaborative study between Saudi Arabia and Lebanon [42, 43].

The regional burden of invasive aspergillosis differs between countries in the GCC, averaging approximately 6 cases per 100,000 individuals (Table 2). The Qatar estimation was the lowest at 0.6/100,000, whereas in Oman, the UAE and Saudi Arabia, the estimated rates were 5.4, 5.4 and 7.6/100,000, respectively [44–47]. The outlier rate of the region was estimated in Kuwait at 16.7/1,000,000, which is considered relatively high in comparison with other GCC countries [46]. This high rate is influenced by the recorded COPD incidence and the high hospitalization rate in Kuwait, which resulted in a perceived overestimation [46]. Moreover, in a 5-year retrospective study conducted in a tertiary hospital in Bahrain, probable IA was reported in 26% of all patients with *Aspergillus*-positive cultures [42]. In Oman, an 8-year retrospective study on children with leukemia reported that invasive fungal infections were diagnosed in 16% of patients, 19% of whom had invasive aspergillosis [48]. Additionally, in Oman, commercial renal transplantation procedures were reported to be complicated with fungal infections, mainly invasive *Aspergillus* infections [49]. In retrospective studies in Saudi Arabia, Invasive Orbital Apex Aspergillosis (IOAA) and Invasive *Aspergillus* Rhinosinusitis (IARS) were associated with subarachnoid hemorrhage and a high level of mortality regardless of immunity compromisation [50, 51]. Additionally, IA was diagnosed in 21% of Chronic Granulomatous Disease (CGD) cases in a 5-year retrospective study in the UAE [52].

Furthermore, ABPA and SAFS are pulmonary disorders induced by hypersensitivity to *Aspergillus* antigens and are associated with asthma, cystic fibrosis and COPD [53]. The regional burdens of ABPA and SAFS averaged approximately 137 and 155 per 100,000 individuals, respectively (Table 2). The estimated rates for ABPA and SAFS are positively proportional to the level of asthma in the population; for example, Kuwait had the highest rate of asthma at 9.5% in the adult population, resulting in the regional highest estimations for ABPA and SAFS at 187 and 246 per 1,000,000, respectively, whereas lower rates of asthma resulted in lower estimations, such as those in Qatar, Oman and the UAE [44–47]. Moreover, in a prospective study conducted on individuals with asthma in Bahrain, *Aspergillus* sensitization was detected in 16% of asthmatics, while 10% were diagnosed with ABPA, which is often accompanied by symptoms such as eczema, allergic rhinitis and conjunctivitis [54].

Common risk factors associated with aspergillosis include uncontrolled diabetes, acute leukemia, hematopoietic stem cell transplantation, solid organ transplant recipients,, neutropenic patients undergoing cancer chemotherapy, prolonged immunosuppressive therapy, tuberculosis, and chronic lung and liver diseases [3, 24]. Additionally, the onset of viral respiratory infections is a major risk factor for aspergillosis development, and a diagnosis is often missed [55]. Indeed, conditions like Influenza-Associated Aspergillosis and Covid-19 Associated Pulmonary Aspergillosis (CAPA) are often developed into Acute Respiratory Distress Syndrome (ARDS) even in immunocompetent individuals as seen in many regional case reports [56–58]. This concomitant infection is thought to be facilitated through the prescription of steroid therapy and IL-6 inhibitors for treating viral respiratory infections, which are predisposing factors for fungal infections such as pulmonary aspergillosis [57, 59].

## Mucormycosis

Mucormycosis, or black fungus, is a spectrum of aggressive filamentous mold infections that are associated with life-threatening conditions in immunocompromised patients [25]. It describes infections caused by molds of the order *Mucorales*, such as *Rhizopus*, *Mucor*, *Rhizomucor*, *Apophysomyces* and *Lichtheimia* (formerly called *Absidia*), among others [25]. It is important to note that zygomycosis was used to describe infections caused by fungi of the phylum Zygomycota, which includes *Mucorales* and *Entomophthorales* [22]. However, after the recent phylogenetic review of the kingdom of fungi, the term zygomycosis became obsolete and was replaced with mucormycosis, referring to *Mucorales*-related infections; basidiobolomycosis, referring to *Basidiobolales*-related infections; and conidiobolomycosis, referring to *Entomophthorales*-related infections [60, 61].

The global estimation of the annual incidence of mucormycosis is more than 200 thousand cases per year [2]. Regionally, the incidence of mucormycosis is similar across available reports, with an average rate of 0.44 cases per 100,000 (Table 2), with most cases involving diabetic patients [44–46]. The most common mucormycosis phenotype in this review was rhino-orbital-cerebral mucormycosis (49%), followed by disseminated mucormycosis at 25%, with *Rhizopus* and *Mucor* being the most commonly reported etiologic agents (35.3% and 14%, respectively), as shown in Table 1.

The clinical presentations of mucormycosis vary depending on the site of infection and the immunological integrity of the host. For example, pulmonary mucormycosis often manifests in immunocompromised individuals through the inhalation of fungal sporangiospores and potentially dissemination into other organs [25]. A case series from Saudi Arabia reported that immunocompromised CGD patients presented with pneumonia onset, pleural effusion and radiographic pulmonary abnormalities, as well as necrotizing granulomatous inflammation due to *Rhizopus* growth [62]. Whereas manifestations of cutaneous mucormycosis often occur in immunocompetent individuals after skin disruption via surgery or traumatic injury [25]. An interesting case report from Kuwait illustrated the gravity of *Apophysomyces elegans* contamination post cosmetic surgery that developed into invasive cutaneous mucormycosis and required a total of 10 sessions of extensive surgical debridement as well as major reconstructive surgery [63]. Similarly, *Mucorales* contamination post trauma may result in complications and dissemination of the fungus into the central nervous system, as seen in necrotizing fasciitis case reports from Saudi Arabia [64–66].

Moreover, rhino-orbital-cerebral (ROC) mucormycosis is another manifestation of the disease that originates mainly in the paranasal sinuses with subsequent invasion of the orbit and brain [67]. It is associated with diabetes and hematological malignancies; in fact, immune dysfunction and defective chemotaxis mediated through uncontrolled diabetes and ketoacidosis are driving factors in the invasion and proliferation of these fungal pathogens [14]. Numerous case reports from the GCC have revealed the associations of ROC mucormycosis with not only uncontrolled diabetes but also with chronic kidney disease, ischemic stroke and liver cirrhosis [68–74].

It is worth noting that gastrointestinal (GIT) infection is another manifestation of mucormycosis, which is a relatively rare condition in adults and affects mainly premature neonates, especially those with low birth weights, or who are receiving immunosuppression therapy [75]. Nevertheless, older patients were reported in the GCC, especially in Saudi Arabia, where 2 cases of GIT mucormycosis were described in children with leukemia (ALL and AML) aged 11 and 12 years, as well as in an adult patient with transfusion-dependent myelodysplastic syndrome [76, 77]. Not unlike aspergillosis, the onset of viral and polymicrobial infections is a major risk factor complicating the clinical manifestations of mucormycosis [78]. Covid-19-associated mucormycosis (CAM) presents a serious healthcare challenge, with a 49% mortality rate globally [78]. A retrospective review of CAM in Oman revealed that mortality reached 60%, and the main reasons for this high rate were ARDS, septic shock and propagation of mucormycosis into the brain [79]. Moreover, the occurrence of bacterial or other fungal infections with mucormycosis has been reported in case reports in the GCC; for example, polymicrobial infections influence misdiagnosis in a pulmonary mucormycosis patient, resulting in delayed diagnosis [80]. Another case report revealed that polymicrobial rhino-cerebral infection was associated with fatal complications resulting from mucormycosis-associated dental extraction [69]. Interestingly, the onset of other fungal infections, such as GIT cryptococcosis, was reported in Saudi Arabia in a ROC mucormycosis patient with uncontrolled diabetes, illustrating the possibility of multiple invasive fungal infections concurrently [81].

Furthermore, the hallmarks of mucormycosis pathogenesis are tissue destruction and angioinvasion [82]. Upon invading the cardiovascular system, the fungal pathogen obstructs normal blood flow, resulting in thrombosis, cardiac ischemia and necrosis of the vascular wall [83]. Indeed, this aggressive infection has a mean estimated number of deaths of 84,000 globally [2]. The WHO estimated the mortality rate to be between 23% and 80% in adult patients [3]. The global mortality rate of ROC mucormycosis is estimated to be 43%, mostly in diabetic patients [67]. The only available GCC reports discussing mucormycosis-associated mortality were in Oman and Saudi Arabia; in a nationwide retrospective multicenter study, mortality rates were reported to be 41.2% and 49%, respectively [84, 85]. However, in another report in the western region of Saudi Arabia, the reported mortality was very high at 73% because of the increased number of immunosuppressed patients visiting the institutional oncology center [86]. However, in another report conducted in the capital city of Riyadh on invasive mucormycosis, the mortality rate was low at 28% because the majority of infections resulted from motor vehicle trauma in immunocompetent individuals [87]. This illustrates the deceiving nature of invasive fungal infections mortality rates, which mainly rely on the risk factors presented by the host. In this review, the mucormycosis mortality rate was 53%, as determined from all related case reports included in this review, as shown in Table 1. Many factors influence the risk of mortality in mucormycosis infection, some of which were encountered in case reports in the GCC, such as lack of suspicion upon admission, onset of hospital-associated infections, and relapse after the preceding antifungal therapy course, among others [67].

### Phaeohyphomycosis

Phaeohyphomycosis is a type of mycosis caused by dematiaceous (pigmented) fungi, particularly species that harbor 1,8 dihydroxynaphthalene (DHN)-melanin in the cell wall [88]. In this review, the terms chromoblastomycosis, chromomycosis and eumycetoma are considered extensions of phaeohyphomycosis to avoid discussing every marginal clinical difference between each phenotype. Moreover, various clinical manifestations may transpire as a result of dematiaceous fungal infection with certain genera linked to specific infections, such as allergic, cutaneous and subcutaneous infections, often caused by *Alternaria*, *Curvularia*, *Bipolaris* and *Exophiala*, whereas invasive cerebral and pulmonary diseases are often caused by *Rhinocladiella (Ramichloridium)* and *Cladophialophora*; the distribution and virulence of these genera depend on the region and the immune integrity of the host [88]. In fact, the WHO does include many of these conditions in the neglected tropical diseases (NTDs) category owing to the associated high morbidity in tropical and subtropical regions, which is overshadowed by underreporting and misdiagnosis [89]. Indeed, there is a dearth of epidemiological reports in the GCC, and the only available report was an 11-year retrospective study from Qatar that reported a 36% prevalence of dematiaceous fungi in diagnosed IFIs, most of which were caused by *Curvularia* [90].

In most cases, this fungal infection is initiated through inoculation from an environmental source as a result of trauma or skin breach, potentially leading to localized cutaneous lesions [91]. However, untreated patients could develop fibrotic and granulomatous infections complicated by sclerotic muriform fungal cell production and micro-abscesses in the affected tissue [91]. Notably, melanin plays significance roles in dematiaceous fungi pathogenicity, as it is suggested to confer protection from cellular oxidative stress and phagocytosis as well as binding to host degradative enzymes, thus preventing fungal plasma membrane disturbance with the combined protection of fungal muriform cellular arrangements [91, 92].

It is postulated that selective pressure imposed by antifungal prophylaxis usage has influenced the manifestation of breakthrough infections such as phaeohyphomycosis in clinical settings and the community [19]. This selective pressure highlights certain pathogens, such as the neurotropic *Rhinocladiella mackenziei*, which is often associated with fatal cerebral infections, even in the GCC, causing cerebral and disseminated phaeohyphomycosis in kidney and liver organ transplantation patients, with an 80% mortality rate [93–95]. Another case of cerebral phaeohyphomycosis, caused by the saprophytic fungus *Fonsecaea*, in combination with COVID-19 pneumonia in a diabetic patient eventually developed sepsis, multiorgan failure, cardiac arrest and death [96]. Interestingly, fatal phaeohyphomycosis has also been reported in immunocompetent individuals with an uncommon invasive subcutaneous infection caused by *Amesia atrobrunnea* (*Chaetomium atrobrunneum*) at surgical sites, which emphasizes the importance of the availability of identification and selective diagnostic methods [97].

## Basidiobolomycosis

Basidiobolomycosis is an inflammatory fungal disease that manifests mainly in subcutaneous tissues, but visceral gastrointestinal (GIB) involvement has often been reported recently [98]. It is caused by the fungus *Basidiobolus*, which belongs to the subphylum *Entomophthoromycotina*, in the order *Basidiobolales*, after recent phylogenic changes, as it used to be included in the phylum *Zygomycota* class *Zygomycetes* [22]. *Basidiobolus* is a saprophytic fungus that is often found in soil and decaying vegetation as well as organic foodstuffs [99]. Transmission through skin-breaking trauma or insect bites is common in subcutaneous infections, whereas in visceral infections, intravenous catheters, ingestion of contaminated foods, intramuscular injections and long-term administration of acid reflux medications are more common [98, 99].

The most common species reported in basidiobolomycosis infections is *B. ranarum*, whose worldwide distribution affects both adult and pediatric populations [98]. Notably, GCC case reports reported another species called *B. omanensis*, which causes fatal disseminated infection in pediatric leukemia (ALL) patients and has been implicated in a GIB case in an adult diabetic Omani patient [100, 101]. This new species has shown elevated resistance to antifungals belonging to the triazole and echinocretin groups; thus, more investigations are needed to determine the propagation of this pathogen [101].

GIB occurs in immunocompromised and immunocompetent individuals with most common presenting symptoms are nonspecific and may include abdominal pain, abdominal mass, constipation, weight loss and prolonged fever [98]. It may stimulate the manifestation of other inflammatory conditions, such as Crohn’s disease, irritable bowel syndrome (IBS), intestinal lymphoma and acute intestinal obstruction [98]. Confusion and misdiagnosis of GIB are relatively common in clinical practice, leading to overlooking the fungal pathogens and the consideration of malignancies, autoimmune conditions or bacterial infections as the causative agent [98, 99]. Indeed, nonspecific symptoms, the concurrence of other GIT-related conditions and the difficulty of obtaining positive fungal cultures all contribute to the difficulty of diagnosing GIB [102–104]. Several case reports in the GCC reiterated this notion, as GIB was initially misdiagnosed and managed as acute appendicitis, colitis, typhlitis, non-Hodgkin lymphoma and adenocarcinoma [102, 104–108]. Interestingly, GIB was reported to mimic mucormycosis angioinvasion, which complicates histopathological analysis, and through molecular analysis only, the diagnosis was ascertained, as mentioned in a case report from Saudi Arabia [109]. Therefore, the diagnosis of basidiobolomycosis requires a high index of suspicion and should be considered in the differential diagnosis of gastroenterological ailments.

## Other mycosis presentations

### Fusariosis

*Fusarium*, the causative agent of fusariosis, is a genetically diverse mold distributed in soil, water and vegetation, notably on palm trees [16, 110]. It is associated with a wide range of disease manifestations ranging from superficial onychomycosis to fatal fungemia due to secondary metabolites production and adventitious sporulation [3, 111, 112]. Additionally, intrinsic antifungal resistance to azoles is another virulence determinant that enhances selective pressure in favor of fusariosis dissemination [3, 16]. The global mortality rate of invasive fusariosis is estimated to be between 43% and 67%, and it affects mainly patients with hematological malignancies [3]. Regionally, a relatively high incidence estimation of fusariosis was reported in the only available epidemiological study in the GCC from Qatar at 1.86 per 100,000 [44]. This high estimation was due to the inclusion of superficial skin and nail infections, which accounted for more than 55% of the reported cases, which in turn made fusariosis one of the most common fungal infections in Qatar [44]. In this review, only two regional case reports were available; both involved immunocompromised patients, one suffering from ALL and the other from ESRD, and despite the invasive nature of the infection, favorable outcomes were achieved [113, 114].

### Cryptococcosis

Cryptococcosis is an opportunistic fungal infection caused by the yeast *Cryptococcus* [3]. It is mostly associated with immunocompromised patients, particularly HIV patients with cellular immunity defects, as well as patients who are receiving immunosuppression therapy [3]. An 11-year retrospective study conducted in Qatar reported that *C. neoformans* was the most common causative agent, followed by *C. laurentii*, mostly causing infections in the CNS, bloodstream and lungs, with a mortality rate of 14% [115]. The global annual incidence for cryptococcal meningitis, which includes HIV patients, is estimated to be more than 200,000 cases; conversely, the regional incidence rates are estimated to be 0.02/100,000 in Oman, 0.1/100,000 in the UAE and 0.43/100,000 in Qatar. These low estimates were attributed to the low HIV rates in the GCC countries [2, 44, 45, 47]. Unfortunately, there are no further epidemiological studies or case reports about cryptococcosis in the GCC available within the boundaries of this review, which is understandable given that the estimated annual incidence of cryptococcal meningitis in Arab League countries (MENA region) is less than 500 cases annually, which is considered low in comparison with other regions in the world [27].

### Isolated incidences

Blastomycosis, histoplasmosis and talaromycosis were found only once in the case reports included in this review. These hyaline fungal infections are often endemic with specific geographical distributions; thus, these infections are considered imported into the GCC region [26]. Blastomycosis is a granulomatous inflammatory infection caused by the fungus *Blastomyces*; it has various clinical manifestations and may mimic other conditions [26, 116]. In a case report from Saudi Arabia, disseminated blastomycosis, supposedly imported from Kentucky, USA, was reported in an immunocompetent patient and manifested as a pleuropulmonary infection mimicking tuberculosis pathophysiology [116]. Histoplasmosis is a generally pulmonary infection caused by the fungus *Histoplasma* and may present as an intracellular parasite in immune cells [26]. The isolated report was from Saudi Arabia and described a patient who underwent left ventricular assist device (LVAD) implantation in India [117]. The onset of disseminated histoplasmosis proceeded after cytomegalovirus (CMV) infection post-transplant, and molecular analysis was paramount in confirming the final diagnosis [117]. Lastly, a supposed Malaysian-imported talaromycosis was reported in Oman in an HIV patient who presented with anorexia, fever and a generalized rash suggestive of chickenpox; however, microbiological and molecular investigations confirmed that the causative agent was *Talaromyces marneffei*, formerly called *Penicillium marneffei* [118].

### Management challenges

We postulate that quality health care, the availability of primary and tertiary care centers even in remote rural areas, as well as the competence of tertiary care centers in terms of facilities and adherence to conventional infection control policies are some of the reasons that kept invasive fungal infections at relatively low incidence rates in the GCC despite the hospitable environment. However, from our point of view, this relatively low incidence is translated into initial confusion and misdiagnosis in many instances that fatal consequences, as we mentioned earlier in this review. This confusion is compounded by the frequent negative fungal cultures and the nonspecific symptoms of many forms of mycosis. In fact, the causative agent of mycosis was confirmed in only about 58% of the case reports included in this review, where in some instances, culture identification resulted in changing the prescribed medication, even changing the class of antifungal to a more appropriate first-line option [80, 119]. Therefore, the availability and implementation of alternative methods of detection are crucial in differential diagnosis, and methods such as molecular analysis (e.g., PCR) and immunological assays (e.g., enzyme immunoassays) actually made a difference in managing fungal infections with negative microbiological cultures, which can lead to favorable outcomes [109, 120].

Antifungal resistance is an emerging phenomenon worldwide, with some clinically relevant fungal species resistant to almost all currently available antifungals [16, 121]. This phenomenon is apparent in GCC reports, with some isolates varying in susceptibility even at the colony level [36, 122]. Unfortunately, fungal pathogens other than *Candida* are often not included in national surveillance of antimicrobial resistance reports, so the rates of antifungal resistance are not available [123]. Indeed, mycosis treatment is challenging when antifungal resistance is considered, with limited therapeutic options and modalities, toxicity associated with the medication, as well as drug-drug interactions, especially in complicated cases [124]. In fact, there has been a dearth of new antifungal regimens with very few additions in the last two decades, which prompt serious action from related international bodies and organizations [84, 124].

The importance of early diagnosis of invasive mold infections cannot be stressed enough, as it is evidently associated with better outcomes [84, 125]. In fact, the poor prognosis associated with CAPA and other infections in immunodeficient patients is often linked to delayed diagnosis and commencement of treatment [58, 84, 126]. Thus, a high degree of suspicion is required for healthcare providers, as being familiar with IFIs, seeking appropriate management and addressing predisposing risk factors are the cornerstones for mitigating the morbidity and mortality of mycosis [63, 84]. Indeed, whenever nonspecific clinical manifestations are presented, detailed clinical examinations and special considerations in patients with predisposing immune deficiencies are suggested [70, 127]. Additionally, suspicion of IFIs in motor vehicle trauma is highly recommended, as a substantial number of cases have been reported in the GCC, given the hospitable environment for fungal inoculation and wound contamination [65, 66, 87]. Notably, appropriate follow-up appointments with previous mycosis patients are important for managing fungal infections, as disease recurrence and dissemination are possible even with a perceived resolution [128]. Furthermore, this notion transcends the responsibility of clinicians to include radiologists to be familiar with typical and atypical radiographic patterns of IFIs, especially in the case of polymicrobial infections. Additionally, clinical microbiologists are required to be familiar with the recent nomenclature of fungal species and even to seek external reference laboratories as resources [19, 24, 39].

### Conclusion and future directions

Fungal pathogens are implicated in a wide range of diseases, ranging from localized cutaneous to life-threatening systemic infections. Despite the low incidence of non-*Candida* mycosis in the GCC, awareness of the gravity of the associated clinical sequalae is needed. Awareness and a high index of suspicion are warranted in successfully managing these infections. Moreover, regular clinical workshops and bulletins discussing invasive mold infections and the consequential manifestations are suggested. Moreover, there is a need for more specific immunological and molecular markers for differential diagnosis, where ruling out fungal infections would be more evidence-based. Additionally, incorporating non-*Candida* mycosis-related antifungal resistance surveys in national surveillance efforts should be enforced, especially when considering the uprise of global mycosis rates.

**Table 1:** Common non-Candida mycosis forms reported in case reports during the timeframe of this review

Disease (*n*)	Reported etiologic agents (*n*,%)	Common clinical manifestations	Commonly reported comorbidities	Mortality rate% (*n*)	References
Aspergillosis (43) Allergic (7, 16.2%) Disseminated (4, 9.3%) Invasive (26, 60.5%) Pulmonary (6, 14%)	*A. fumigatus* (14, 33%) *A. flavus* (12, 28%) *A. niger* (5, 12%) *A. terreus* (3, 7%) *A. nidulans* (1, 2%)	**Allergic** Severe chest tightness, dyspnea, cough, asthma exacerbation, nasal obstruction, rhinorrhea, repeated sneezing episodes, headache, eosinophilia **Disseminated** Loss of weight, poor appetite, fever **Invasive** Fever, vomiting, headache, neutropenia, syncopal attacks, encephalitis, fatigue, weight loss, axillary lymphadenopathy, nasal obstruction, pulmonary embolism, hemoptysis **Pulmonary** Fever, sore throat, cough, mucopurulent expectoration, dyspnea, polyarthralgia, red eyes, pneumonia, pleural effusion, cytopenia, hypoxia	Asthma, ESRD, LOT HPSCT, Polymicrobial infection, Covid-19, diabetes, hypertension	37% (*n* = 16)	[56–58, 120, 128–153]
Mucormycosis (51) Cutaneous (5, 10%) Disseminated (13, 25%) GIT (2, 4%) Pulmonary (5, 10%) Rhino-orbital-cerebral (25, 49%) Genital Tract (1, 2%)	*Rhizopus* (18, 35.3%) *Mucor* (7, 14%) *Apophysomyces* (3, 6%) *Lichtheimia* (1, 2%)	**Cutaneous** Fever, swelling, skin inflammation and necrosis **Disseminated** Tissue necrosis, headache, shortness of breath, night sweats, cough, chest pain, intermittent fever, tachycardia **GIT** Abdominal pain, nausea, vomiting, distended abdomen, diarrhea, perforated hollow viscus **Pulmonary** Fever, cough, hemoptysis, weight loss, crepitations, pulmonary abscess, pneumonia, pleural effusion, enlarged liver and spleen **Rhino-orbital-cerebral** Sinusitis, orbital cellulitis, facial pain, periorbital swelling, chemosis, fever, proptosis, ophthalmoplegia, confusion, complete or partial loss of vision/hearing, numbness, ptosis, nasal obstruction **Genital Tract** Vaginal bleeding, acute villitis, tissue necrosis	Diabetes, Covid-19, Trauma. LOT HPSCT, Polymicrobial infection, CGD, ALL, AML	53% (*n* = 27)	[62–66, 68–73, 76, 77, 79–81, 119, 126, 154–169]
Phaeohyphomycosis (16) Cerebral (10, 63%) Disseminated (4, 25%) Subcutaneous (2, 12%)	*Rhinocladiella mackenziei* (5, 31%) *Neoscytalidium dimidiatum* (2,13%) *Fonsecaea* (2,13%)	**Cerebral** Nausea, vomiting, lethargy, paresis, radicular pain, coordination impairment, hemiplegia, Seizure **Disseminated** Epigastric pain, nausea and anorexia, fever, neutropenia, chest pain **Subcutaneous** Acute inflammation, abscess, swelling	LOT HPSCT, leukemia, CGD	69% (*n* = 11)	[94–97, 170]
Basidiobolomycosis (25) Gastrointestinal (20, 80%) Disseminated (2, 8%) Colorectal (3, 12%)	*Basidiobolus* (8, 32%)	**Gastrointestinal** Fever, abdominal pain, abdominal mass, weight loss, abdominal distension, anorexia, and diarrhea **Disseminated** Fever, abdominal distention, generalized lymphadenopathy, hepatosplenomegaly, cellulitis **Colorectal** Constipation, abdominal pain, Rectal bleeding, weight loss, ulcers, abdominal distention, vomiting, fever, eosinophilia	Diabetes, Abdominal mass	24% (*n* = 6)	[100–109, 171–174]

ALL, Acute Lymphocytic Leukemia; AML, Acute Myeloid Leukemia; CGD, Chronic Granulomatous Disease; ESRD, End Stage Renal Disease; GIT, Gastrointestinal tract; HPSCT, Hematopoietic stem cell transplant; LOT, Live Organ Transplant

**Table 2:** Average incidence estimations for non-Candida mycosis forms in the GCC countries

Disease	Average incidence estimation/100,000	References
Invasive Aspergillosis	6	[43–47, 84]
Chronic Pulmonary Aspergillosis	11.3	
ABPA	137	
SAFS	155	
Mucormycosis	0.44	

ABPA, Allergic Bronchopulmonary Aspergillosis; SAFS, Severe Asthma with Fungal Sensitization

## Data Availability

No datasets were generated or analysed during the current study.

## Declarations

Clinical Trial.
